# Progress in Surgery

**Published:** 1894-12-08

**Authors:** 


### Progress in Surgery.

### SURGERY OF THE VERMIFORM APPENDIX.

**Appendicitis and Appendicular Colic.**—Dr. Rushmore's experience makes him conclude[1] that appendicitis is a surgical disease from the beginning, and that, all things considered, appendicectomy offers the best chance, immediate and remote, for the patient. The operation should be done at the earliest possible moment. Dr. G. R. Fowler considers[2] (1) that appendicitis results primarily from circulatory and nervous disturbances which greatly lower the resistance of the part, and that the vascular and nervous disturbances are due either to immediate torsion of the meso-appendix or chronic progressive hyperplasia of the same. (2) That the nature of the inflammation in the given case will be catarrhal, fibrinous, purulent, a combination of the above-named, or interstitial. These, in turn, will depend upon the degree of circulatory and nervous disturbance, and upon the nature of the micro-organisms present. (3) It can now be certainly shown that, in the given cases of acute appendicitis, this initial attack is resultant from sudden torsion, and is not the first warning of a chronic infective meso-appendicitis, with progressive trophic lesions of the appendix. (4) Weighing the chances of general infection of the peritoneum, or of the system at large engendered by delay, with those of infection from a properly conducted operation, the decision, from a pathological standpoint, must be in favour of early operation. He thus classifies lesions of the vermiform appendix:—

1. Hyperplasia; general anomalies of formation.
2. Circulation disturbances; oligæmia; hyperæmia.
3. Non-specific inflammation—(*a*) Catarrhal, (*b*) purulent, (*c*) fibrinous, (*d*) combinations of (*a*), (*b*), and (*c*); (*e*) interstitial.
4. Specific inflammation—(*a*) Tubercular, (*b*) typhoid.
5. Progressive nutrition disturbances—(*a*) Consecutive hyperplasia of any or all of the tissues, (*b*) neoplasms (carcinoma, endothelioma, sarcoma).
6. Regressive nutrition disturbances; atrophic changes involving any or all the coats from deficient vascular or nervous supply.
7. Foreign bodies, such as enteroliths or accidental substances.
8. Parasites; the various intestinal micro-organisms.

Mayo Robson says[3] an infective appendical inflammation may pursue one of several courses, or several courses together: (1) Local adhesive peritonitis by direct irritation of the peritoneal envelope, leading to adhesions of the appendix to neighbouring parts. (2) Abscess, either sub-peritoneal, due to lymphatic infection, or within the tube, due to ulceration of mucous membrane, the lumen of the tube having become blocked. The abscess, once formed, may burrow between the layers of the mesentery of the tube, forming an extra peritoneal abscess, or may burst into contiguous viscera, or into the general peritoneal cavity, producing perforative general peritonitis. (3) Inflammation may extend along the lymph channels to the cæcum, producing a true typhlitis or perityphlitis, which may or may not end in suppuration. (4) Infectious thrombosis of the vessels; if of the single artery, leading to gangrene of the appendix and acute peritonitis; if of the veins, leading to septicæmia or pyæmia, or possibly to abscess of the liver. (5) General peritonitis by direct extension without perforation, producing paralysis of bowel and symptoms of acute intestinal obstruction. (6) Violent colic, due to the irritation of the muscular coat and its violent contractions in trying to force the contents of the tube, whether fæcal or other concretion, or pus or mucus, through the contracted orifice into the cœcum. (7) Stricture, due to the healing of an ulcer and accumulation of mucus in the distal end of the tube, producing periodical attacks of colic.

Dr. J. M. Barton reports[4] the results of fourteen operations for appendicitis in which the appendix was not removed. These were all cases of ruptured appendix with circumscribed abscess, with no general peritonitis and no symptoms of obstruction. All on whom he had operated in this manner had recovered, and none that he was aware of had had any trouble with the retained appendix since. The mortality had been much greater when he had removed the appendix. He thought it likely, in these cases, that the opening from the appendix into the intestine was closed early in the attack. If it were not firmly closed, the pus would never have broken through the walls of the appendix, or, having broken through, the resulting abscess would not have increased in size, but would have emptied itself through the appendix into the bowel. When the appendix is still unruptured, or when it has ruptured and general peritonitis has occurred, or when obstruction is present, he removes it. Dr. J. A. Wyeth maintains[5] that the danger to life which makes appendicitis strictly a surgical disease is peritonitis, and this may occur with, or without, perforation. He does not believe it is possible to diagnose by symptoms whether perforation is or is not about to occur. This does not imply that by a study of symptoms one cannot make out perforation in a good proportion of cases where this has occurred. The death rate of 364 recorded cases was 18 per cent. It ought easily to be brought down to 5 per cent. by avoiding unnecessary delay, and if all cases were operated upon within the first twenty-four hours by skilful surgeons, it would be reduced to 1 or 2 per cent. The surgeon might be mistaken now and then, but the error would be on the side of safety. Opening the peritoneal cavity was not a dangerous procedure, and the likelihood of hernia occurring at a small incision was slight.

McBurney describes[6] a new method of operating in certain cases of appendicitis, which promises a more perfect result than usual as regards the strength of the abdominal wall. The incision in the skin is an oblique one, crossing a line from the anterior iliac spine to the umbilicus nearly at a right angle, about one inch from the iliac spine, and is so situated that its upper third lies above that line. The section of the external oblique muscle and aponeurosis should correspond, great care being taken to separate these tissues in the same line, not cutting any fibres across. The edges in the wound of the external oblique are strongly pulled apart with retractors, displaying a considerable expanse of the internal oblique muscle, the fibres of which cross somewhat obliquely. These fibres, and those of the transversalis muscle can then be separated with a blunt instrument in a line parallel to their course. The edges of this deeper wound are next separated with blunt retractors, thus exposing well the transversalis fascia, which is then divided in the same line. Last of all the section of the peritoneum is made. Two sets of retractors must be used, one holding open the superficial wound from side to side, the other separating the edges of the deeper wound from above downwards. The muscular and tendinous fibres being separated and not divided, muscular action will rather tend to approximate the edges of the wound. Hæmorrhage is slight. The transversalis fascia can easily be completely sutured. The operation requires more time and more assistance than the usual one. It is not suitable for cases accompanied by suppuration about the appendix, nor for non-suppurative cases which require a large intra-abdominal dissection during operation. The author has performed this operation on four patients in the intervals between attacks of recurrent appendicitis.

Dr. Morris insists on the necessity for immediate operation in cases of appendicitis, and maintains[7] that a long incision is not necessary in cases that are operated upon at the outset of the inflammation, nor, as a rule, in cases operated on in the interval, and there will be no hernias and no permanent scars if the surgeon will accept as a standard an incision one inch-and-a-half in length, the divided structures of the abdominal wall being united separately with fine catgut. The abdominal scar cannot be seen at the end of a few months. The death-rate has been *nil* in cases in which suppuration was not present, and diagnostic accuracy has so increased that it is unusual to find pus. Dr. Morris states[8] that when the inch-and-a-half abdominal incision is employed, patients leave the hospital at the end of a week and a half. The time required for repair increases in proportion to the length of the incision. Dr. Porter has analysed[9] 448 cases collected from various sources, with the view of indicating the results of various modes of treatment. Of 151 cases treated by removal of the appendix during an attack of inflammation 29 died, 19·7 per cent.; of 14 treated by removal of appendix during quiescence one died from sepsis, 7·14 per cent.; of 188 cases treated by incision and drainage 34 died, 18·18 per cent.; and of 95 treated without operation 13 died, 13·68 per cent. The average mortality of the total number of cases was 17·23 per cent. Dr. Porter does not advocate removal of the appendix in cases of abscess, unless it be found in the cavity; if adhesions exist, he regards it as safer to leave it alone. Dr. J. Swain believes[10] that in cases of suppurative appendicitis it would be far wiser not attempt the removal of the appendix than to run any risk of opening the general peritoneal cavity during the separation of adhesions, for the purpose of getting at the appendix.

Dr. Jonas reports[11] a case of enterolith mistaken first for appendicitis and then for carcinoma. The patient had a sudden onset of pain which localised itself over the ileo-cæcal region, elevated temperature, accelerated pulse, constipation, and pain aggravated on pressure near McBurney's point. After ten days a tumour was discovered, which on operation was thought to be a case of irremovable carcinoma of the bowel. About one month later, however, the woman passed per rectum an enterolith about as large as a lemon, black as tar, and hard as stone. She then rapidly improved. Dr. L. Brunton and Mr. W. Cheyne quoted[12] a case of intestinal obstruction, due to constriction of the bowel, after appendicitis. The patient, aged 35, had suffered from attacks of appendicitis for some years. For thirteen days before operation he had attacks of acute griping pain in the abdomen, the bowels, however, being open throughout, though imperfectly. Thirty-six hours before operation there was complete obstruction, followed by collapse, and fæcal vomiting. The diagnosis was narrowing of the bowel from gradual contraction of fibrous tissue, resulting from repeated inflammatory attacks, and this was confirmed at the operation. When the abdomen was opened the appendix which encircled the cæcal valve was removed, and the adhesions were cut or torn through until the contents of the small intestine could be readily squeezed into the large. He made an uninterrupted recovery. H. C. Cameron reports[13] a case in which an inflamed vermiform appendix had formed a communication with the bladder. Wind, blood, and pus used to escape *per urethram*. On opening the abdomen the appendix was found attached to the bladder by what ought to have been its free end. It was ligatured and cut out, only the two ends being left at their points of attachment. Writing[14] on the subject of appendicular colic, Mr. Jessop concludes that (1) The vermiform appendix is liable to partial occlusion of its canal from various causes, some of which are permanent, while others are transient; (2) The symptoms by which such incomplete obstruction is to be recognized are those of "appendicular colic"; (3) In cases of recurring appendicular colic, and especially if there be at the same time an increasing severity, our practice should be to recommend the removal of the appendix.

[1] Annals of Surgery, May, 1894, p. 574. [2] Ibidem, April, 1894, p. 475. [3] Lancet, June 30th, 1894, p. 1,608. [4] Annals of Surgery, July, 1894, p. 84. [5] Med. Rev., N.Y., April 28th, 1894, and Med. Week, April 20th, 1894. [6] Annals of Surgery, July, 1894; and Brit. Med. Journ., August 11th, 1894. [7] Med. Chronicle, April, 1894, p. 53. [8] Op. Cit. [9] Edin. Med. Journ., May, 1894. [10] Bristol Med. Chirurg. Journ., March, 1894 [11] Med. Record, N.Y., March 3rd, 1894, p. 267. [12] Lancet, May 5th, 1894, p. 1,135. [13] Glasgow Med. Journ., August, 1894, p. 143. [14] Brit. Med. Journ., March 24th, 1894, p. 627.

